# Use of bone-SPECT/CT and Na[^18^F]F-PET/CT in hyperparathyroidism

**DOI:** 10.3389/fnume.2025.1565906

**Published:** 2025-04-16

**Authors:** Wouter van der Bruggen, Bernard F. Bulten

**Affiliations:** Department of Nuclear Medicine, Slingeland Hospital, Doetinchem and Streekziekenhuis Koningin Beatrix, Winterswijk, Netherlands

**Keywords:** hyperparathyroidism, bone-SPECT/CT, sodium fluoride PET/CT, brown tumor, benign bone disease, bone-related complications

## Abstract

Hyperparathyroidism disrupts the balance of physiological bone formation and resorption by upregulating osteoclast activity. This leads to hypercalcemia, resulting in osteoporosis and eventually the formation of “brown tumors.” Currently used radiological and nuclear medicine imaging for primary hyperparathyroidism face challenges in accurately diagnosing bone-related complications. Molecular bone imaging techniques routinely consist of bone scintigraphy, with possible addition of bone-SPECT/CT. Recently, renewed interest has emerged in the use of Na[^18^F]F-PET/CT. Both applications are highly sensitive to *in vivo* osteoblast activity. However, the latter technique offers improved spatial resolution and sensitivity, as well as shorter incubation and faster scanning. This article summarizes current limitations and potential improvements in bone-SPECT/CT and Na[^18^F]F-PET/CT imaging in selected patients with hyperparathyroidism, compared to other relevant techniques and clinical parameters.

## Introduction

Currently used radiological and nuclear medicine imaging for primary hyperparathyroidism (PHPT) face challenges in accurately diagnosing bone-related complications. Limited sensitivity and specificity of radiological techniques and of planar nuclear imaging techniques hamper the early accurate staging of bone involvement in these patients. This article describes the potential role and limitations of advanced molecular and multimodality imaging techniques to identify bone involvement in PHPT, especially for bone-SPECT/CT and Na[^18^F]F-PET/CT.

## Calcium homeostasis and bone abnormalities in hyperparathyroidism

Calcium homeostasis is a tightly regulated process aiming to maintain stable calcium blood levels (1). The parathyroid glands play a central role by secreting parathyroid hormone (PTH) in response to low serum calcium levels. PTH increases calcium release from bones and enhances renal calcium reabsorption. PHPT, which is characterized by excessive PTH production, increases osteoclast activity and therefore bone resorption, leading to hypercalcemia (1). This is reflected in biochemical marker measurements as well as dynamic histomorphometry (2). Hypercalcemia influences the (neuro)muscular, skeletal, cardiovascular, renal, and gastrointestinal systems (3). Prolonged increased osteoclast activity will at first lead to a systemic decrease in the amount of normal bone tissue, i.e., osteoporosis (4), but may eventually result in lesional destruction of trabeculae and degeneration of the fibrovascular tissue, ultimately leading to cysts. These changes, known as osteitis fibrosa cystica, are a hallmark of persisting severe hyperparathyroidism (4–6). The newly originated cysts then become populated with macrophages, which may contain the iron-storage complex hemosiderin. Hemosiderin can be observed microscopically as a brown pigment (7). Deposition of hemosiderin, combined with the ingrowth of fibrovascular tissue into the dissecting osteitis cavities, may eventually form so-called brown tumors (5). Osteoporosis, osteitis fibrosa cystica, and brown tumors all weaken the bone, placing patients at risk for pathological fractures.

Though rare, as hypercalcemia is nowadays readily detected by routine calcium screening, brown tumors are an important clinical manifestation of uncontrolled hyperparathyroidism and their characteristics have been extensively documented in the literature (8–10). Brown tumors are more prevalent in primary hyperparathyroidism than in secondary forms and can affect various bones, including the ribs, pelvis, and facial bones (8, 9, 11). These lesions mimic malignancies radiographically and histologically, posing diagnostic challenges (1, 12). Recognizing the early signs of PHPT-related bone changes and understanding the pathophysiology of osteitis fibrosa cystica and brown tumors—along with their appearance across various imaging modalities—can facilitate timely intervention and help prevent irreversible skeletal complications.

## Nuclear medicine techniques to image bone turnover

In healthy individuals, osteoblasts and osteoclasts work in a continuous feedback cycle of bone formation and resorption, called bone turnover (13). Several diseases, either originating from within or outside of the bone, may disrupt this balance. Bone diseases can be categorized as osteoclastic, osteoblastic, or a combination of both. In most benign bone and joint conditions, the osteoblastic component predominates (13). As described above, PHPT results in increased osteoclast activation and bone resorption (5). Bone remodeling in reaction to these lesions involves osteoblast activation (14).

Molecular bone imaging predominantly consists of bone scintigraphy, with the possible addition of cross-sectional single photon emission tomography with overlay of computed tomography (bone-SPECT/CT) (15). Recently, renewed interest in the use of sodium fluoride positron emission tomography (PET) with integrated CT (Na[^18^F]F-PET/CT) has been expressed (13, 16, 17). Both techniques are highly sensitive to detect osteoblast activity through the injection of radiopharmaceuticals (18, 19). While the uptake mechanisms of these radiopharmaceuticals differ slightly, they are influenced by regional bone perfusion and ultimately bind to the surface of hydroxyapatite crystals via chemisorption (20, 21). Na[^18^F]F-PET/CT boasts rapid uptake in bone, faster scanning, and improved spatial resolution over bone-SPECT/CT, and the newest PET/CT scanners allow for minimal radiation exposure (13, 19). Both techniques have the capability to detect clinically relevant early changes in bone turnover, with proven additional value in the management of patients with benign and malignant bone disease (15–17). Where compared to conventional morphological bone imaging (i.e., x-ray, dual-energy x-ray absorptiometry, and standalone CT), these molecular imaging techniques identify bone changes several months before structural anatomical changes can be detected (15, 16, 21, 22).

### Planar bone scintigraphy

Planar bone scintigraphy has been the cornerstone of nuclear medicine bone imaging for many decades. It is a known imaging modality to depict osteoblastic activity, dependent on vascularization, blood pool, and bone turnover, with the ability to detect generalized or focal metabolic changes in bone turnover (15, 23).

In several metabolic bone diseases, including PHPT, osteomalacia, and renal osteodystrophy, key findings on bone scintigraphy include increased tracer accumulation in the axial skeleton, long bones, periarticular zones, skull, mandibulae, and sternum (24). Still, in mild cases of metabolic changes and in asymptomatic patients, the sensitivity of these findings is poor, while the specificity in differentiating PHPT from other metabolic bone diseases, such as renal osteodystrophy, is also low (24). Therefore, bone scintigraphy is not routinely used to diagnose PHPT (15, 24).

Secondary hyperparathyroidism (SHPT), consisting of excessive secretion of PTH by the parathyroid glands in response to hypocalcemia, is the main cause of a metabolic superscan (23). When compared to malignant causes of a superscan, a metabolic superscan typically presents with homogeneous, symmetrical increased bone uptake, often featuring symmetrical uptake in the mandible and calvarium, as well as enhanced periarticular uptake and costochondral beading (23). However, differentiating between benign and malignant uptake with complete certainty cannot be achieved through planar scintigraphy alone.

Diagnosis becomes more challenging in severe or protracted cases of the disease due to the formation of brown tumors. Three-phase bone scintigraphy is highly sensitive in detecting these lesions by pinpointing accumulations of ^99m^Tc-methyl diphosphonate (MDP). Bone scintigraphy outperforms ^99m^Tc-sestamibi scan in detecting brown tumors (10).

The possibility of a brown tumor should be included in the differential diagnosis when focal accumulation of ^99m^Tc-MDP is observed, especially in patients with diffuse abnormal uptake suggestive of metabolic bone disease (9, 25). However, a focal hotspot – with or without generalized abnormal uptake – is not specific to brown tumors, as fissures or fractures can also present in patients with metabolic bone disease (11, 24). In addition, brown tumors are well known to mimic malignant bone lesions on planar bone scintigraphy and plain radiographs, making accurate differentiation essential (5, 9, 23, 26).

Correlation to other molecular or radiological findings and biochemical results (i.e., PTH) improves specificity (10).

### Bone-SPECT/CT

Although planar bone scintigraphy is an impactful technique in case of clinically insufficiently explained hypercalcemia (23, 24), adding bone-SPECT/CT to the planar scan significantly improves the diagnostic value of the study (13, 15). SPECT improves spatial resolution, contrast, and localization, especially in complex three-dimensional (3D) structures, such as the skull, spine, pelvis, and hind- and midfoot (13). The main advantage, however, lies in characterizing focal lesions and in differentiating concurrent co-morbidity, such as bone metastases (27).

The addition of (low-dose) CT adds morphological characteristics to the uptake on SPECT and allows for attenuation correction, thus improving the quality of the SPECT images. Morphologically, brown tumors are depicted as well-defined, primarily lytic and often expansile lesions, predominantly occurring in the mandible, clavicle, ribs, and pelvis (28, 29). They can present with intra-focal calcifications, a sclerotic rim, and fluid-fluid levels (27). Attenuation values are in the range of blood and fibrous tissue, in the range of approximately +13 to +75 (28, 30). CT may also reveal additional pathology in or outside of the bones, such as hematoma or soft tissue swelling.

Although bone-SPECT/CT adds cost per patient, with exact expenses and reimbursement varying by region or country, its cost-effectiveness for metabolic bone disease remains unstudied (31). Furthermore, radiation exposure must be considered, as bone-SPECT/CT adds 0.2–2 mSv per bed position, depending on the region of interest (32). Despite these factors, integrating bone-SPECT/CT with planar scintigraphy is recommended for patients with suspected PHPT (15).

### Sodium fluoride PET/CT (Na[^18^F]F-PET/CT)

Sodium fluoride labeled with radioactive fluorine-18 (Na[^18^F]F) precisely depicts *in vivo* blood flow into the bone and subsequent systemic and focal bone remodeling by imaging osteoblasts (21). It is a smaller compound compared to ^99m^Tc-MDP and offers advantages in pharmacokinetics, including fast blood clearance, high first-pass extraction, low non-specific protein binding, and efficient bone uptake via chemisorption (20). This allows Na[^18^F]F-PET to swiftly detect changes in osteoblast activity shortly after the onset of many (benign) bone diseases (13).

In comparison to the abovementioned bone scintigraphy protocols, including bone-SPECT/CT, Na[^18^F]F-PET/CT offers 3D and cross-sectional imaging of the whole skeleton, with shorter incubation and acquisition times (19, 33). Na[^18^F]F-PET/CT provides improved spatial resolution and more robust quantification abilities (19, 34). Moreover, PET/CT hardware typically comprises new generation CT possibilities, leading to high-quality CT images, superior iterative metal artifact reduction (iMAR), and low radiation exposure. Typically, radiation exposure of whole-body Na[^18^F]F-PET/CT is estimated at approximately 1.9 mSv for PET acquisitions and 2–3 mSv for low-dose CT (13, 19, 31, 34). These favorable characteristics, combined with recent shortages of ^99m^Tc, have led to renewed interest in Na[^18^F]F-PET/CT (19, 21). At the same time, Na[^18^F]F-PET/CT increases costs per patient, has limited availability, and may not be reimbursed in certain countries or regions (16).

Na[^18^F]F-PET/CT has already been proven to be safe and effective in different groups of patients with benign bone disease (35, 36). In patients with primary hyperparathyroidism, this imaging modality might be particularly useful, as it provides insights into both generalized and focal metabolic bone changes, while conveniently depicting the whole skeleton.

A metabolic superscan pattern on Na[^18^F]F-PET/CT is characterized by diffusely increased homogenous Na[^18^F]F-uptake in the axial and appendicular skeleton, including the calvarium and distal extremities, with decreased background activity in the soft tissues (37).

Given that PHPT is often diagnosed early, one might expect extensive literature describing this phenomenon on Na[^18^F]F-PET/CT in detail. However, research remains relatively scarce, with more focus on the less frequently encountered osteitis fibrosis cystica and brown tumors (14, 21, 25, 37). In contrast, the metabolic superscan pattern is well documented in ^99m^Tc-bone scintigraphy (15, 23, 24, 38, 39).

### Other imaging techniques for characterizing lesions in PHPT

^99m^Tc-MIBI SPECT/CT and ^18^F-fluorocholine PET/CT and ultrasound are well-recognized imaging procedures for the detection of primary parathyroid adenoma (40, 41). Of these modalities, ^18^F-fluorocholine PET/CT is considered the most sensitive technique, especially in small adenomas (25, 40). It is also capable of identifying brown tumors, with improved accuracy over ^99m^Tc-MIBI or bone scintigraphy (40, 41). However, ^18^F-fluorocholine PET/CT does not adequately reflect bone turnover changes and expresses a significantly lower target-to-background ratio for bone pathology in comparison with Na[^18^F]F-PET/CT and bone-SPECT/CT. Furthermore, costs for ^18^F-fluorocholine PET/CT are higher.

Since brown tumors share morphological characteristics with other lytic lesions (i.e., multiple myeloma or osteolytic metastases), conventional CT imaging alone cannot be used to confidently differentiate in all cases.

[^18^F]FDG is a non-invasive imaging marker for increased macrophage activity and glucose transporters (GLUT1 and GLUT3). It likely also targets other cellular changes commonly associated with inflammation and infection (42). In the literature about patients with benign bone and bone marrow disease, [^18^F]FDG-PET/CT is known to detect intra-articular changes, as well as focal or systemic bone marrow changes (43–45). As described earlier, macrophages are involved in hemosiderin deposition in brown tumors, which may explain the increased [^18^F]FDG-uptake (7). The sensitivity of [^18^F]FDG-PET/CT for detecting brown tumors is reported to be higher than that of ^99m^Tc-MIBI scintigraphy (46) and may detect brown tumors in unexpected localizations, such as in the mandible and maxillary bone (12, 47). In addition, its high sensitivity for these lesions, combined with routine whole-body imaging, often enables the detection of unanticipated brown tumors (48). However, a downside on the use of [^18^F]FDG -PET/CT is its limited specificity, as [^18^F]FDG-avid osteolytic lesions may also represent metastases of a solid tumor, such as pulmonary cancer (25).

## Future perspectives

Similarities in the uptake mechanism of Na[^18^F]F-PET/CT and bone scintigraphy, combined with the known superiority in imaging characteristics of the former, suggest that Na[^18^F]F-PET/CT will perform well in revealing the metabolic superscan pattern. This capability, however, still has to be confirmed in a structured series of patients with PHPT. Ideally, a cost-effectiveness study should validate the use of Na[^18^F]F-PET/CT over bone scintigraphy.

The activity of brown tumors on Na[^18^F]F-PET/CT correlates with the renal glomerular filtration rate and serum calcium levels (14). In addition, the total metabolically active bone volume, as quantified by Na[^18^F]F-PET/CT, is associated with serum PTH and alkaline phosphatase levels. This could even reflect clinical outcomes, such as the duration of postoperative intravenous calcium replacement and extended hospitalization after parathyroidectomy (14). According to Graf et al., the increased bone turnover in brown tumors can persist for an extended period after PHPT treatment (14). This means Na[^18^F]F-PET/CT might be an appropriate imaging choice to evaluate not only patients with active PHPT, but also patients with a history of the disease. On the contrary, Jacquet-Francillon et al. report that after parathyroidectomy, the uptake of radiotracers in brown tumor lesions is reversible, emphasizing the dynamic nature of these lesions in response to treatment for hyperparathyroidism (25). Additional research is required to address this discrepancy.

In the near future, the addition of dynamic Na[^18^F]F-PET/CT might deliver quantitative data on bone perfusion, blood pool, and bone turnover, possibly further improving the evaluation of hyperparathyroidism-related bone disease (21, 49). In addition, further evidence is needed to more conclusively state whether Na[^18^F]F-PET/CT adds clinically relevant value over bone-SPECT/CT in patients suspect of PHPT. The limited current literature suggests that patients with previously indeterminate lesions in particular might benefit.

A multidisciplinary approach, incorporating clinical evaluation, biochemical testing, pathological examination, radiological imaging, and advanced molecular imaging including bone-SPECT/CT and Na[^18^F]F-PET/CT, is pivotal to differentiate brown tumors from bone metastases and prevent misdiagnosis, especially in patients with osteolytic lesions and hyperparathyroidism (26, 50).

## Conclusions

Planar bone scintigraphy is a valuable tool for investigating hypercalcemia in patients with suspected metabolic bone disorders. It reveals generalized increased bone turnover, while simultaneously (15, 23, 24) detecting focal bone disease (24). However, its limited specificity requires additional bone-SPECT/CT for adequate characterization of focal lesions (27, 29).

As Na[^18^F]F-PET/CT offers superior image acquisition and quality, in theory, this seems to be a better imaging modality for the evaluation of these patients. However, the literature on this topic is still limited, and availability and reimbursement of the modality is not always guaranteed. Further research is needed to gather evidence on the use of Na[^18^F]F-PET/CT for this indication and assess its cost-effectiveness.

## References

[1] Bilezikian JP. Primary hyperparathyroidism. J Clin Endocrinol Metab. (2018) 103(11):3993–4004. 10.1210/jc.2018-01225 30060226 PMC6182311

[2] Costa AG Bilezikian JP. Bone turnover markers in primary hyperparathyroidism. J Clin Densitom. (2013) 16(1):22–7. 10.1016/j.jocd.2012.11.004 23374737 PMC3577420

[3] Carroll MF Schade DS. A practical approach to hypercalcemia. Am Fam Physician. (2003) 67(9):1959–66. 12751658

[4] Jowsey J. Bone histology and hyperparathyroidism. Clin Endocrinol Metab. (1974) 3(2):267–84. 10.1016/S0300-595X(74)80010-1 4611665

[5] Carsote M Ciobica ML Sima OC Valea A Bondor CI Geleriu A Brown tumors: the hidden face of primary and renal hyperparathyroidism amid real-life settings. J Clin Med. (2024) 13(13):3847. 10.3390/jcm13133847 38999413 PMC11242279

[6] Misiorowski W Czajka-Oraniec I Kochman M Zgliczyński W Bilezikian JP. Osteitis fibrosa cystica-a forgotten radiological feature of primary hyperparathyroidism. Endocrine. (2017) 58(2):380–5. 10.1007/s12020-017-1414-2 28900835 PMC5671544

[7] Braumann A Wulfhekel U Düllmann J Nielsen P. Iron overload of the bone marrow by trimethylhexanoyl-ferrocene in rats. Acta Anat (Basel). (1992) 144(4):285–95. 10.1159/000147318 1414192

[8] Pai M Park CH Kim BS Chung YS Park HB. Multiple brown tumors in parathyroid carcinoma mimicking metastatic bone disease. Clin Nucl Med. (1997) 22(10):691–4. 10.1097/00003072-199710000-00006 9343725

[9] Meydan N Barutca S Guney E Boylu S Savk O Culhaci N Brown tumors mimicking bone metastases. J Natl Med Assoc. (2006) 98(6):950–3. 16775919 PMC2569361

[10] Meng Z Zhu M He Q Tian W Zhang Y Jia Q Clinical implications of brown tumor uptake in whole-body 99mTc-sestamibi scans for primary hyperparathyroidism. Nucl Med Commun. (2011) 32(8):708–15. 10.1097/MNM.0b013e328347b582 21613966

[11] Jordan KG Telepak RJ Spaeth J. Detection of hypervascular brown tumors on three-phase bone scan. J Nucl Med. (1993) 34(12):2188–90. 8254409

[12] Selvi F Cakarer S Tanakol R Guler SD Keskin C. Brown tumour of the maxilla and mandible: a rare complication of tertiary hyperparathyroidism. Dentomaxillofac Radiol. (2009) 38(1):53–8. 10.1259/dmfr/81694583 19114425

[13] van der Bruggen W. PET/CT & SPECT/CT in benign bone disease, thesis (Doctor of philosophy). University of Twente, Enschede (2023).

[14] Graf C Huellner M Tschopp O Bode-Lesniewska B Schmid C. (18)F-NaF-PET/CT in patients with primary hyperparathyroidism and brown tumors. J Bone Miner Metab. (2020) 38(3):299–309. 10.1007/s00774-019-01059-z 31760503

[15] Van den Wyngaert T Strobel K Kampen WU Kuwert T van der Bruggen W Mohan HK The EANM practice guidelines for bone scintigraphy. Eur J Nucl Med Mol Imaging. (2016) 43(9):1723–38. 10.1007/s00259-016-3415-4 27262701 PMC4932135

[16] Beheshti M Mottaghy FM Paycha F Behrendt FFF Van den Wyngaert T Fogelman I (18)F-NaF PET/CT: eANM procedure guidelines for bone imaging. Eur J Nucl Med Mol Imaging. (2015) 42(11):1767–77. 10.1007/s00259-015-3138-y 26201825

[17] Rohren EM Macapinlac HA. Spectrum of benign bone conditions on NaF-PET. Semin Nucl Med. (2017) 47(4):392–6. 10.1053/j.semnuclmed.2017.02.008 28583278

[18] van der Bruggen W Hirschmann MT Strobel K Kampen WU Kuwert T Gnanasegaran G SPECT/CT in the postoperative painful knee. Semin Nucl Med. (2018) 48(5):439–53. 10.1053/j.semnuclmed.2018.05.003 30193650

[19] van der Bruggen W Hagelstein-Rotman M de Geus-Oei LF Smit F Dijkstra PDS Appelman-Dijkstra NM Quantifying skeletal burden in fibrous dysplasia using sodium fluoride PET/CT. Eur J Nucl Med Mol Imaging. (2020) 47(6):1527–37. 10.1007/s00259-019-04657-1 31875244

[20] Ahuja K Sotoudeh H Galgano SJ Singh R Gupta N Gaddamanugu S (18)F-sodium fluoride PET: history, technical feasibility, mechanism of action, normal biodistribution, and diagnostic performance in bone metastasis detection compared with other imaging modalities. J Nucl Med Technol. (2020) 48(1):9–16. 10.2967/jnmt.119.234336 31811067

[21] Park PSU Raynor WY Sun Y Werner TJ Rajapakse CS Alavi A. (18)F-sodium fluoride PET as a diagnostic modality for metabolic, autoimmune, and osteogenic bone disorders: cellular mechanisms and clinical applications. Int J Mol Sci. (2021) 22(12):6504. 10.3390/ijms22126504 34204387 PMC8234710

[22] van der Bruggen W de Geus-Oei LF Bosmans B Slart R Lima TVM Bhure U Review of the role of bone-SPECT/CT in tarsal coalitions. Nucl Med Commun. (2023) 44(2):115–30. 10.1097/MNM.0000000000001643 36630216

[23] Askari E Shakeri S Roustaei H Fotouhi M Sadeghi R Harsini S Superscan pattern on bone scintigraphy: a comprehensive review. Diagnostics (Basel). (2024) 14(19):2229. 10.3390/diagnostics14192229 39410633 PMC11475626

[24] Ryan PJ Fogelman I. Bone scintigraphy in metabolic bone disease. Semin Nucl Med. (1997) 27(3):291–305. 10.1016/S0001-2998(97)80030-X 9224668

[25] Jacquet-Francillon N Prevot N. Brown tumors in nuclear medicine: a systematic review. Ann Nucl Med. (2023) 37(5):255–70. 10.1007/s12149-023-01832-1 36933117

[26] Paramita RD Rahardjo P. Multiple brown tumor in late adolescence mimicking bone metastasis: a challenging case report. Radiol Case Rep. (2024) 19(10):4266–72. 10.1016/j.radcr.2024.07.008 39135674 PMC11318562

[27] Sun L Peng R. The value of integration of bone scan and targeted SPECT/CT in diagnosis of primary hyperparathyroidism with multiple bone brown tumor. Skeletal Radiol. (2023) 52(12):2505–11. 10.1007/s00256-023-04361-0 37227482

[28] Chew FS Huang-Hellinger F. Brown tumor. AJR Am J Roentgenol. (1993) 160(4):752. 10.2214/ajr.160.4.8456657 8456657

[29] Xie C Tsakok M Taylor N Partington K. Imaging of brown tumours: a pictorial review. Insights Imaging. (2019) 10(1):75. 10.1186/s13244-019-0757-z 31359305 PMC6663953

[30] Fosbinder RO. D. Chapter 17 computed tomography. In: Sabatini P, editor. Essentials of Radiologic Science. Baltimore, MD: Lippincott Williams & Wilkins (2011). p. 263.

[31] Van den Wyngaert T Palli SR Imhoff RJ Hirschmann MT. Cost-effectiveness of bone SPECT/CT in painful total knee arthroplasty. J Nucl Med. (2018) 59(11):1742–50. 10.2967/jnumed.117.205567 29602816

[32] Biswas D Bible JE Bohan M Simpson AK Whang PG Grauer JN. Radiation exposure from musculoskeletal computerized tomographic scans. J Bone Joint Surg Am. (2009) 91(8):1882–9. 10.2106/JBJS.H.01199 19651945

[33] Nogueira-Lima E Alves T Etchebehere E. (18)F-fluoride PET/CT-updates. Semin Nucl Med. (2024) 54(6):951–65. 10.1053/j.semnuclmed.2024.09.005 39393951

[34] de Ruiter RD Zwama J Raijmakers P Yaqub M Burchell GL Boellaard R Validation of quantitative [(18)F]NaF PET uptake parameters in bone diseases: a systematic review. Ann Nucl Med. (2025) 39(2):98–149. 10.1007/s12149-024-01991-9 39729191 PMC11799077

[35] Langsteger W Balogova S Huchet V Beheshti M Paycha F Egrot C Fluorocholine (18F) and sodium fluoride (18F) PET/CT in the detection of prostate cancer: prospective comparison of diagnostic performance determined by masked Reading. Q J Nucl Med Mol Imaging. (2011) 55(4):448–57. 21738117

[36] Beheshti M. (18)F-sodium fluoride PET/CT and PET/MR imaging of bone and joint disorders. PET Clin. (2018) 13(4):477–90. 10.1016/j.cpet.2018.05.004 30219183

[37] Edamadaka Y Parghane RV Basu S. Complimentary role of [18F]FDG and [18F]NaF-PET/CT in evaluating synchronous thyroid carcinoma and parathyroid adenoma with brown tumors. World J Nucl Med. (2024) 23(3):220–4. 10.1055/s-0044-1787732 39170840 PMC11335386

[38] Kovacsne A Kozon I Bentestuen M Zacho HD. Frequency of superscan on bone scintigraphy: a systematic review. Clin Physiol Funct Imaging. (2023) 43(5):297–304. 10.1111/cpf.12821 37070619

[39] Liu Y. Super-superscan on a bone scintigraphy. Clin Nucl Med. (2011) 36(3):227–8. 10.1097/RLU.0b013e318208f503 21285685

[40] Beheshti M Hehenwarter L Paymani Z Rendl G Imamovic L Rettenbacher R (18)F-fluorocholine PET/CT in the assessment of primary hyperparathyroidism compared with (99m)Tc-MIBI or (99m)Tc-tetrofosmin SPECT/CT: a prospective dual-centre study in 100 patients. Eur J Nucl Med Mol Imaging. (2018) 45(10):1762–71. 10.1007/s00259-018-3980-9 29516131 PMC6097754

[41] Zhang-Yin J Gaujoux S Delbot T Gauthé M Talbot JN. 18F-fluorocholine PET/CT imaging of brown tumors in a patient with severe primary hyperparathyroidism. Clin Nucl Med. (2019) 44(12):971–4. 10.1097/RLU.0000000000002814 31652163

[42] Abikhzer G Treglia G Pelletier-Galarneau M Buscombe J Chiti A Dibble EH EANM/SNMMI guideline/procedure standard for [^18^F]FDG hybrid PET use in infection and inflammation in adults v2.0. Eur J Nucl Med Mol Imaging. (2025) 52(2):510–38. 10.1007/s00259-024-06915-3 39387894 PMC11732780

[43] Burke CJ Walter WR Gaddam S Pham H Babb JS Sanger J Correlation of benign incidental findings seen on whole-body PET-CT with knee MRI: patterns of (18)F-FDG avidity, intra-articular pathology, and bone marrow edema lesions. Skeletal Radiol. (2018) 47(12):1651–60. 10.1007/s00256-018-3001-x 29931417

[44] Inoue K Goto R Okada K Kinomura S Fukuda H. A bone marrow F-18 FDG uptake exceeding the liver uptake may indicate bone marrow hyperactivity. Ann Nucl Med. (2009) 23(7):643–9. 10.1007/s12149-009-0286-9 19629627

[45] van der Bruggen W Glaudemans A Vellenga E Slart R. PET in benign bone marrow disorders. Semin Nucl Med. (2017) 47(4):397–407. 10.1053/j.semnuclmed.2017.02.006 28583279

[46] Gahier Penhoat M Drui D Ansquer C Mirallie E Maugars Y Guillot P. Contribution of 18-FDG PET/CT to brown tumor detection in a patient with primary hyperparathyroidism. Joint Bone Spine. (2017) 84(2):209–12. 10.1016/j.jbspin.2016.06.007 27726929

[47] Yamaga E Fujioka T Asakage T Miura K Tateishi U. 18F-FDG-detected brown tumor confined to the maxillary bone with parathyroid adenoma. Clin Nucl Med. (2022) 47(3):236–8. 10.1097/RLU.0000000000003897 34560690

[48] Geysen A Van Laere K Verscuren R. Detection of unexpected brown tumors due to hyperparathyroidism diagnosed by 18F-FDG PET/CT. Clin Nucl Med. (2021) 46(1):e16–7. 10.1097/RLU.0000000000003380 33181751

[49] Puri T Frost ML Cook GJ Blake GM. [(18)F] sodium fluoride PET kinetic parameters in bone imaging. Tomography. (2021) 7(4):843–54. 10.3390/tomography7040071 34941643 PMC8708178

[50] Parikh P Shetty S Rodrigues G Bhat SN. Brown tumour mimicking skeletal metastasis. BMJ Case Rep. (2021) 14(7):e243478. 10.1136/bcr-2021-243478 34257125 PMC8278892

